# Deceleration of probe beam by stage bias potential improves resolution of serial block-face scanning electron microscopic images

**DOI:** 10.1186/s40679-016-0025-y

**Published:** 2016-09-15

**Authors:** James C. Bouwer, Thomas J. Deerinck, Eric Bushong, Vadim Astakhov, Ranjan Ramachandra, Steven T. Peltier, Mark H. Ellisman

**Affiliations:** National Center for Microscopy and Imaging Research, University of California at San Diego, BSB 1000, 9500 Gilman Dr., La Jolla, CA 92093-0608 USA

**Keywords:** SBEM, Deceleration, SEM, Volume reconstruction, Serial section, Cerebellum, Backscatter electron detector

## Abstract

Serial block-face scanning electron microscopy (SBEM) is quickly becoming an important imaging tool to explore three-dimensional biological structure across spatial scales. At probe-beam-electron energies of 2.0 keV or lower, the axial resolution should improve, because there is less primary electron penetration into the block face. More specifically, at these lower energies, the interaction volume is much smaller, and therefore, surface detail is more highly resolved. However, the backscattered electron yield for metal contrast agents and the backscattered electron detector sensitivity are both sub-optimal at these lower energies, thus negating the gain in axial resolution. We found that the application of a negative voltage (reversal potential) applied to a modified SBEM stage creates a tunable electric field at the sample. This field can be used to decrease the probe-beam-landing energy and, at the same time, alter the trajectory of the signal to increase the signal collected by the detector. With decelerated low landing-energy electrons, we observed that the probe-beam-electron-penetration depth was reduced to less than 30 nm in epoxy-embedded biological specimens. Concurrently, a large increase in recorded signal occurred due to the re-acceleration of BSEs in the bias field towards the objective pole piece where the detector is located. By tuning the bias field, we were able to manipulate the trajectories of the  primary and secondary electrons, enabling the spatial discrimination of these signals using an advanced ring-type BSE detector configuration or a standard monolithic BSE detector coupled with a blocking aperture.

## Background

Serial block-face scanning electron microscopy (SBEM) has proved to be a remarkable technique for imaging at moderate lateral and axial resolution (approximately 10 and 40 nm, respectively) and across large fields of view spanning many hundreds of microns of sample. This technique is elucidating processes, where selectively stained cells within large fields of view can be found [1–3] and small details can be followed across multi-scale dimensions. It has been especially useful in neuron tracking across large distances [4]. One SBEM study that tracked mitophagy events from neurons in their neighboring astrocytes [5], in particular, has fundamentally changed how scientists look at the role played by astrocytes in glaucoma.

The SBEM platform is comprised of an ultramicrotome embedded within the scanning electron microscope (SEM) chamber. Originally conceived by Leighton [6], use of this imaging technique has become mainstream, because of an automated platform to collect 3D volumes developed by Denk and Horstmann [7]. With SBEM, the embedded microtome removes ~30–80-nm-thick sections from the sample block mounted on a fixed rivet. With each “slice”, the electron-beam raster scans the newly exposed block face, and the BSEs are collected to create an image. A 3D volume is obtained by stacking the images of the block face obtained after each microtomy cycle.

The platform improved by Denk and Horstmann was commercialized by Gatan, Inc., as the 3View system. The latter has been integrated into multiple SEM platforms across manufacturers and features full computer automation. In 2015, a competing SBEM platform, the Teneo VS, was released by FEI Company.

The focused ion beam milling technique (FIB-SEM) is analogous to SBEM, but the block face is vaporized by bombardment by heavy ions to remove subsequent layers of sample [8, 9]. As with SBEM, FIB-SEM backscattered electron images are collected after each bombardment and stacked to form a 3D reconstruction.

In spite of their different methods for removing the exposed block face, SBEM and FIB-SBEM use SEM backscattered electron imaging, and thus, improvements in preparing specimens and detectors are germane to both technologies. In this study, we concentrated on determining the theoretical and experimental factors in BSE collection critical to further improvements, particularly using primary beam reversal potentials to improve detection efficiency and image resolution.

It has long been known that applying a negative potential to the sample reduces beam-landing energies and, thereby, reduces beam damage to the specimen and improves surface detail. This understanding informed the design of the low-energy electron microscope (LEEM) [10, 11]. LEEM has supported complex studies in materials science [12–15]. However, in LEEM, the sample is generally irradiated with a flood beam and uses complex instrumentation of magnetic sector plates to separate electrons leaving the sample from the primary beam. There have been only a few studies of stage bias in a conventional SEM and, specifically, its relevance to imaging biological samples [9, 16, 17]. For imaging biological samples, secondary electron (SE) signal generally provides unsatisfactory contrast. BSE imaging, however, provides a clear contrast between the heavy-metal stain and unstained structures [7]. It was been pointed out that, although beam deceleration in SBEM improves the contrast and resolution of the BSE image, it also leads to severe image artifacts whose cause is unclear [9]. We believe that, to enable more widespread use of the beam-deceleration techniques in SBEM, a comprehensive study of its advantages and pitfalls is needed. This manuscript tries to address these issues.

## Methods

### Specimen mounting

Small (1 mm × 1 mm × 0.5 mm) pieces of resin-embedded tissues were mounted on aluminum specimen pins (Gatan, Inc., Pleasanton, CA, USA) using cyanoacrylic glue and precision trimmed with a glass knife to a rectangle approximately 0.5 mm × 1.0 mm, so that tissue was exposed on all four sides. Silver paint (Ted Pella, Inc., Redding, CA, USA, http://www.tedpella.com) was used to electrically ground the edges of the tissue block to the aluminum pin, taking care not to get paint on the block face or edges of the embedded tissue to be sectioned. The entire specimen was then sputter coated with a thin layer of gold/palladium. After the top layer of gold/palladium block was removed by the ultramicrotome, the tissue morphology became visible by BSE imaging. The remaining coating on the edges of the block served to reduce charging and did not interfere with imaging.

### Implementing the stage bias potential on two SEM/3View platforms

The initial experiments using stage biasing to achieve deceleration were performed on a FEI Quanta 200 FEG scanning electron microscope on loan from FEI. The Quanta FEG was equipped with a high-precision dc power supply to apply a negative-bias potential to the sample. The Quanta FEG was also equipped with a 3View system from Gatan, Inc. The backscatter electron detectors (BSDs) included a monolithic backscatter electron diode detector from Gatan, Inc., as well as a concentric backscatter (CBS) detector from FEI.

These two backscatter detectors are both approximately 9 mm in diameter and include a 1-mm central hole to allow the probe beam to pass through. The two detectors were also positioned to subtend a similar solid angle for BSEs.

The monolithic BSE detector from Gatan, Inc., was read out, though a single output into the amplification and digitization circuitry. The FEI CBS detector, on the other hand, reads signals from the concentric rings of active regions through four individually configurable amplifiers. The geometry of this device allows for the spatial differentiation of signal (i.e., signal reaching the inner vs. the outer rings is read out separately). When used in conjunction with a biasing field, this device is advantageous, as it allows for the ability to spatially discriminate SE from BSE signals (e.g., to eliminate SE signal contamination when performing BSE imaging).

A similar strategy for signal differentiation was implemented on a Zeiss Sigma variable-pressure SEM platform equipped with a Gatan 3View system and a monolithic BSE detector. Gatan, Inc., also provided a high-precision dc power supply to bias the sample potential with a stable and accurate negative voltage. In this case, we used a custom-made aperture to block unwanted SEs from striking the BSE detector and polluting the BSE signal. The SE-blocking aperture was machined to our specifications as determined by the gun-acceleration potential, the negative-bias potential, and the working distance. The blocking aperture was attached to the grounded shielding of the Gatan BSE detector using silver paint (Ted Pella, Inc.) and suspended above the detector by thin wires.

**Fig. 1:**
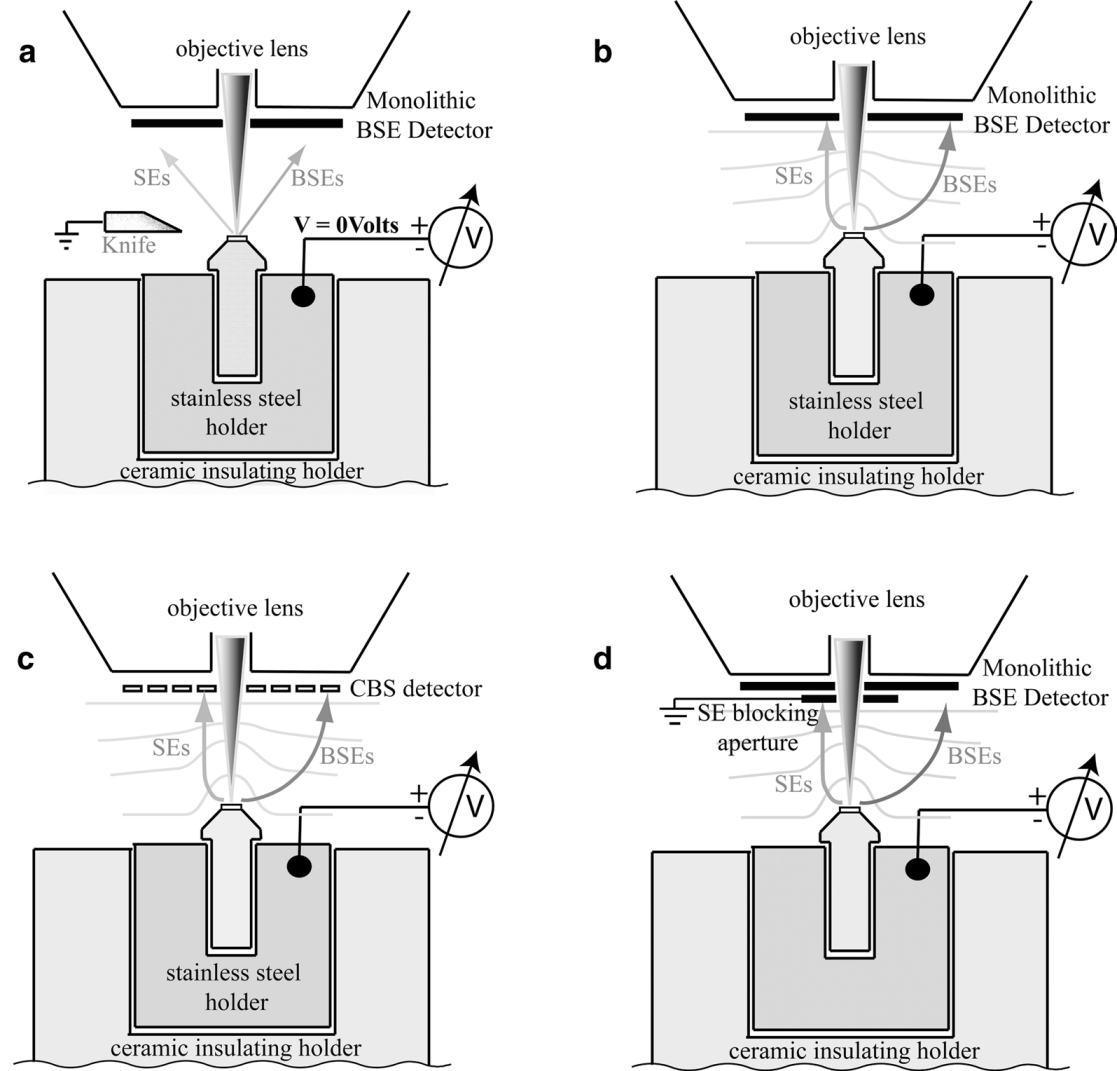
Scaled diagram of SBEM stage-biasing geometry for various configurations of SBEM and SBEM with deceleration. The aluminum pin with mounted sample sits inside a stainless steel holder. The holder is attached to a high-stability power supply to provide negative-biasing potential. The biased sample, sample pin and stainless steel holder are insulated in a machined ceramic holder. The diamond knife is held at ground potential. Since diamond is a poor conductor, no arcing occurs during cutting at voltages lower than 3.5 keV. The sample working distance is maintained at 6.6 mm during imaging on the FEI Quanta 200 and 8.8 mm on the Zeiss Sigma. **a** Setup for the conventional SBEM without deceleration. The SEs and BSEs propagate in straight lines without the effect of electric fields. **b** Layout for deceleration with the monolithic BSE detector. The SE and BSE signals are convolved in the detector, causing artifacts. **c** The SEs are collected in the central ring of the CBS detector. Since each ring has its own amplifier, the SEs and BSEs can be separated to provide pure BSE images. **d** Configuration used on a Zeiss Sigma. SEs are collected by an SE-blocking aperture connected to ground, while a pure BSE signal is collected by the Gatan monolithic BSE detector

To apply a bias voltage to the sample, we implemented modifications to electrically isolate the microtome and the sample. The 3View ultramicrotomes on both platforms were modified by machining an insulating ceramic holder that mounts where a metal holder usually sits. The sample holder and sample pin were electrically conductive and connected to a highly stable, adjustable dc voltage source. A variable-bias voltage of 0 to −5000 V could then be applied to the sample. Figure 1 shows the setup for the various modes of operation: without deceleration using a monolithic BSE detector, with deceleration using the monolithic BSE detector, with deceleration using the CBS detector, and with deceleration operating with a monolithic BSE detector and an SE-blocking aperture. Illustrations of the SE and BSE trajectories as affected by the biasing electric fields are shown along with the illustrations of the equipotential lines during sample biasing.

**Fig. 2:**
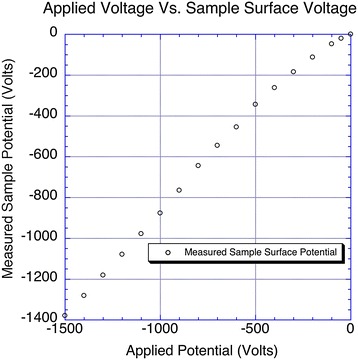
Plot of the potential applied to the pin versus the measured surface potential. Above −500 V, the surface potential measured shows an approximately 12 % drop in the measured voltage versus applied voltage

The metal-stained samples used in these experiments are geometrically complex: They are composed of part plastic insulator and part heavy-metal-stained tissue with a multitude of densities. As a result, accurate calculations of sample permittivity (the ability of a substance to store electrical energy) are quite difficult to model and, thus, need to be determined experimentally. To do this, we used a special gold electrode tacked to the plastic-embedded sample surface with silver paint to verify the voltage on the sample surface. To confirm that the voltage source was outputting the correct values, we connected an electrode to the sample-mounting pin. The output voltage measured at the aluminum sample pin was confirmed to be accurate to within 99.2 % of the requested output voltage. Once we verified the output voltage from the source, we tested the voltage at the surface of the plastic-embedded block as a function of voltage applied to the aluminum pin (the measured surface potential as a function of applied voltage for each block is presented in Fig. 2), and the electron-landing energy was then matched with the appropriate electron-landing-energy, penetration depth, and cutting thickness settings.

## Results and discussion

### Factors affecting BSE image resolution in the SBEM

Scattering of incident electrons in the SEM is governed principally by the probe-beam-landing energy and composition of the scattering substrate. The penetration depth of the probe-beam electrons into the sample (electron range), the lateral scattering of the probe beam and BSEs, and the thickness of the ultramicrotome cuts all limit resolution in the SBEM. The electron range for carbon is ~60 and 180 nm for beam energy of 1.5 and 3 keV, respectively, according to the Kanaya and Okayama range equation [18].

**Fig. 3:**
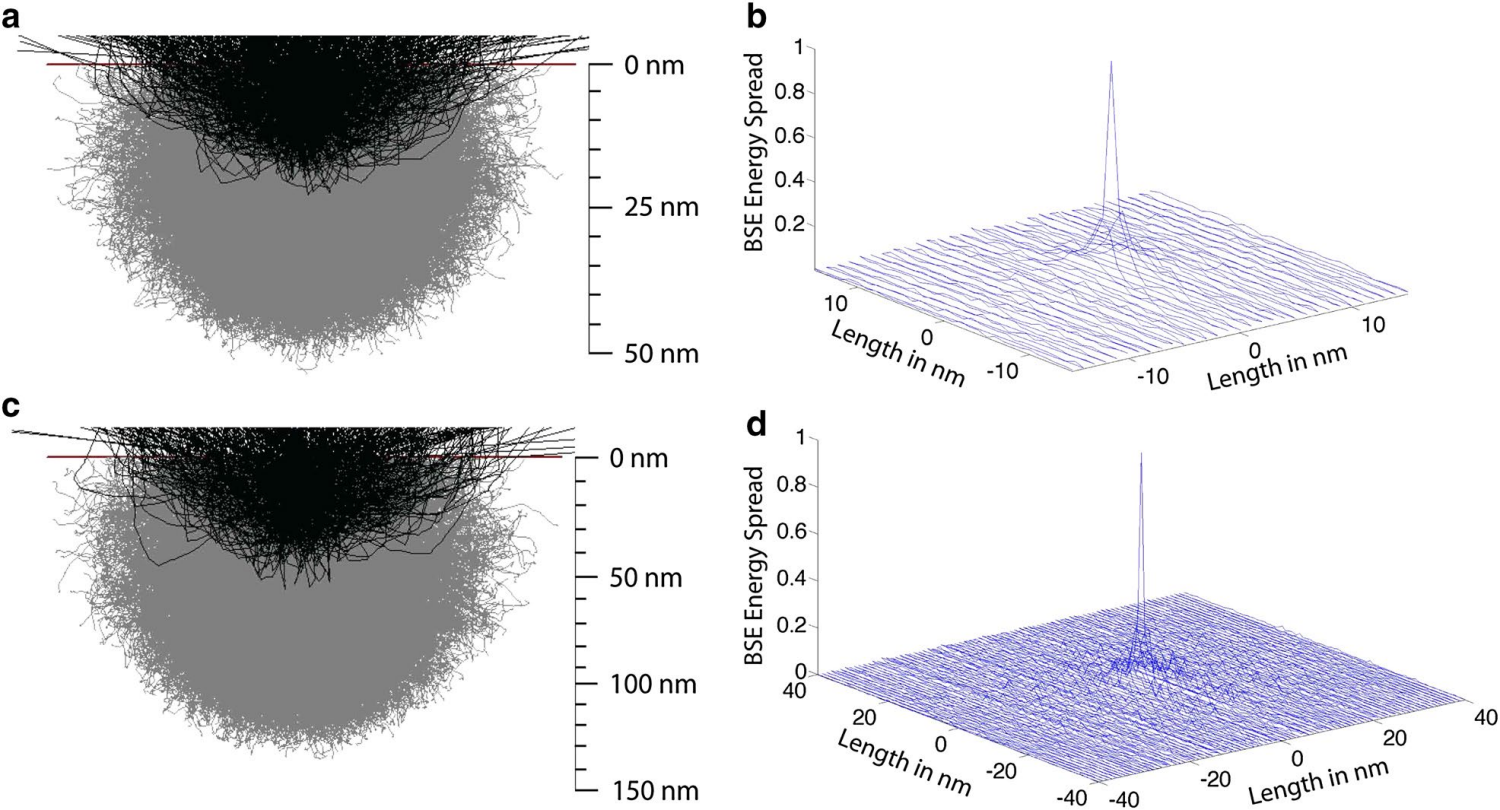
Monte Carlo simulation of electron–beam interaction in a carbon substrate. **a** Penetration depth of a 1.5-keV electron beam. **b** Line scan of a BSE signal profile for the 1.5-keV electron beam. **c** Penetration depth of a 3-keV electron beam. **d** Line scan of a BSE signal profile for the 3-keV electron beam. The simulations in **a** and **c** were performed for 10,000 electrons, with the electrons emerging as BSE shown in a darker shade. The simulations in **b** and **d** were performed at a pixel size of 1 nm for 1 million electrons. An infinitesimally thin-electron beam was assumed in all the simulations

We performed the Monte Carlo simulations of electron–beam interaction with a carbon substrate similar to those detailed in [19]. In the simulations, we used 1 million electrons. Figure 3a, c shows the electron-trajectory plot of a subset of electrons (10,000 electrons) used in the simulation at 1.5 and 3 keV. The BSEs that escape from the surface after successive scattering are plotted in black, and the incident beam electrons that do not escape, i.e., the incident electrons that get deposited in the carbon substrate, are plotted in gray. The electron range predicted by the simulation is slightly lower than the Kanaya and Okayama range: ~50 and 130 nm for beam energies of 1.5 and 3 keV, respectively.

**Fig. 4:**
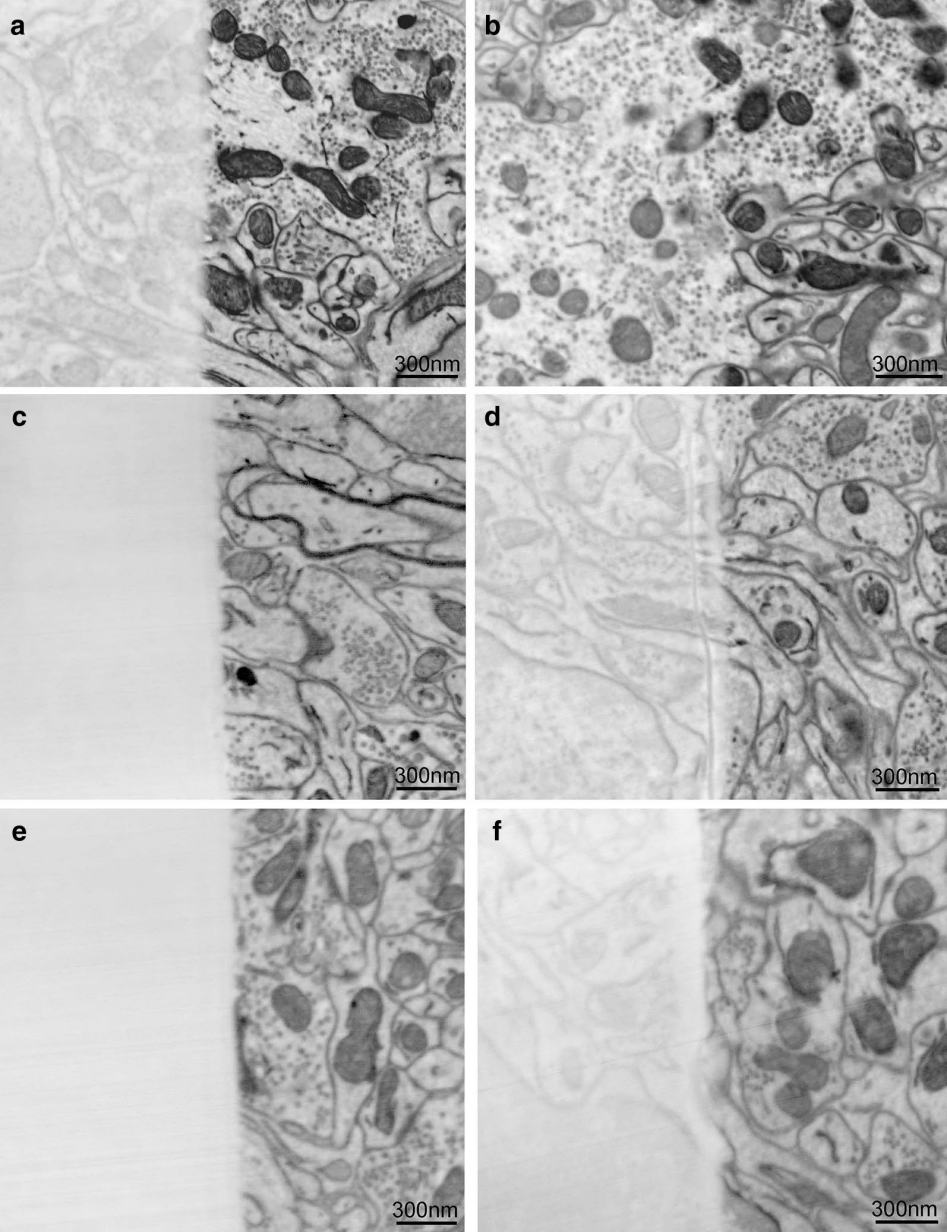
Determination of BSE penetration depth. Sections of 50-nm (**a**, **c**, **e**) or 30-nm (**b**, **d**, **f**) thickness of pure Durcupan resin were overlaid on one half of the tissue block (visible on the *left half* of each image). At 3.0 keV, a significant number of BSEs emanated from beneath the 50- and 30-nm-thick sections (**a**, **b**). At 2.0-keV accelerating voltage, almost no signal was observed from below the 50-nm section (**c**) while some was noted from below a 30-nm-thick section (**d**). At 1.7 keV, no signal was observed from below a 50-nm section (**e**), while very little was observed from below a 30-nm-thick section (**f**). Note that the decrease in signal to noise as the acceleration voltage is lowered

**Fig. 5:**
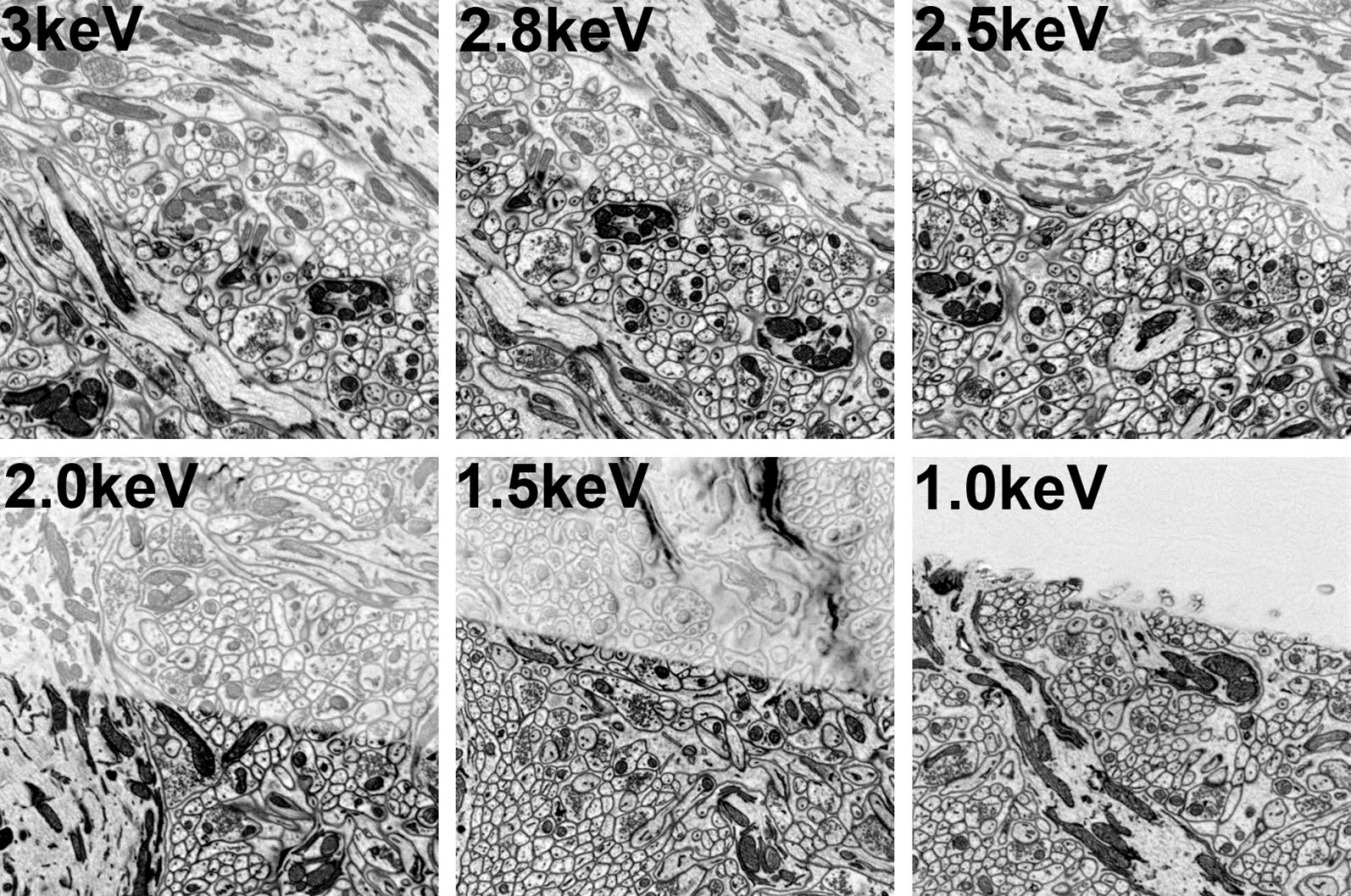
Electron-penetration depth as a function of probe-beam-landing energy modulated via a decelerating biasing potential. A 30-nm-thick blank plastic section was placed on top of the cerebellum block sample measured in Fig. 4. Images were then collected on the FEI CBS detector with the central ring turned off. The 30-nm section runs diagonally across the middle of the image. Images are inverted, so *white* shows no backscattering signal. All images were acquired with 3-keV column-electron energy and deceleration appropriate to achieve the electron-landing energy  shown in each panel. At a landing energy between 1.5 and 1 keV, the electron-penetration depth drops below 30 nm

The escape depth of the BSEs is much smaller than the electron range and, therefore, provides information from a much shallower region of the sample. Figure 3a, b shows that the escape depth of the BSE is ~20 and 40 nm, respectively, for the 1.5- and 3-keV electron beams. The escape depth of the BSEs is generally ~0.2–0.3 times the electron range [20], which is consistent with our simulation and corroborates very well with our experimental data (Figs. 4, 5).

Another, important parameter that determines the ability to resolve fine features in BSE imaging is the lateral-energy spread of the BSEs, i.e., how far the BSEs emerge from the incident-beam impact point and the fraction of the incident beam energy they have. The lateral-energy spread was calculated by dividing the area around the beam impact point into pixels of size 1 and 2 nm for the 1.5- and 3-keV beams, respectively, and the total integrated BSE signal emanating from each pixel was computed as the summation of the energy of all the BSEs from the particular pixel. The lateral-energy spread was measured in nm as the distance from the incident-beam impact point that contains 50 % of the total BSE energy. It was found to be 11 nm and 36 nm for the 1.5- and 3-keV beams, respectively (Fig. 3b, d).

The only way to physically improve the axial resolution of electron scattering from a theoretical basis is to lower the energy of the probe-beam electrons. However, below 2 keV, beam control is problematic as the electromagnetic fields used to steer the electrons down the column are less stable, and the less-energetic primary beam is more susceptible to external electric and magnetic interference leading to degraded resolution [21]. In addition, at lower accelerating voltages, chromatic aberration can degrade resolution as well. Chromatic aberration is defined as follows:

1 $$\delta_{\text{c}} = C_{\text{c}} \left( {\frac{\Delta E}{{E_{0} }}} \right)\alpha$$

where *δ*_c_ is the disk of least confusion, *C*_c_ is the chromatic aberration coefficient, *α* is the aperture semi-angle, *E*_0_ is the accelerating energy, and Δ*E* is the width of the energy distribution of beam electrons [22].

### Decreasing primary beam-landing energy improves axial resolution

We originally used an FEI Quanta 200 ESEM equipped with a Gatan 3View for our SBEM imaging. The optimal performance for this SBEM was achieved at lower landing energies (down to 1.7 keV). At these energies, we achieved a smaller interaction volume in the sample that resulted in improved axial resolution and less energy deposition on the specimen. As a result, we were able to cut finer sections. However, at these lower energies, our probe was also subject increased chromatic aberration effects and we experienced decreased BSE detector sensitivity.

We initially performed landing-energy experiments by placing a 50- and a 30-nm-thick blank section of Durcupan resin cut on a Leica UC EM6 ultramicrotome over a biological sample, then imaging through the layer. As we varied the acceleration voltage, we looked for signal emanating from the layers below. Figure 4 shows a panel of images of the 50-nm-blank Durcupan resin overlays on the left and 30-nm-blank section overlays on the right imaged at 3.0, 2.0, and 1.7 keV. At 3.0 keV, a great deal of signal is observed from below the epoxy layer for both 50- and 30-nm-blank sections. After stepping down to 2.0 keV, the interaction volume is reduced. As expected, we see much less signal coming from the region beneath the 30-nm-blank section and just about no signal coming from the region beneath the 50-nm section. As the acceleration voltage was decreased to 1.7 keV, very few BSEs originating from below 30 nm were detected, demonstrating that we effectively reduced our penetration depth.

However, image quality was degraded due to decreased detector sensitivity. These low-energy-loss BSEs arose from elastically scattered or nearly elastically scattered electrons. Small energy losses are due to interactions with the atomic nuclei of the sample stain, and they backscatter with nearly the same energy as the probe electrons [21, 23]. Since most solid-state backscatter detectors are built from silicon photo-diode circuitry that have a thin-surface passivation layer and metalized electrodes, low-energy BSEs do not deposit much ionization charge in the active region of the diode and are difficult to detect above inherent detector noise.

### Testing the impact of beam deceleration on SBEM resolution

To achieve the low electron-landing energies required for thinner penetration while avoiding detector sensitivity issues, we decelerated the probe-beam electrons at the final stage of imaging after the objective lens with the use of a negative-bias voltage applied at the sample stage.

We repeated the same overlay experiments as shown in Fig. 4, but this time, we kept the accelerating voltage fixed at 3.0 keV and adjusted the negative-bias voltage to produce a final landing energy between 3.0 and 1.0 keV. Figure 5 shows the images of the plastic-embedded sample with a 30-nm-blank section overlay across the surface at various landing energies, achieved by adjusting the negative reversal potential. It is clear from the BSE signal-depth retrieval images that we reduced our penetration depth and retained good image quality and signal to noise even at extremely low landing energies. For 30-nm ultramicrotome sectioning, a landing energy of between 1.0 and 1.5 keV was optimum. Electrons striking the BSE detector did so with an energy nearly matching the 3-keV instrument accelerating voltage and with high signal to noise: on the order of 20× greater signal can be achieved at a similar landing energy without the use of the negative-bias voltage.

Repeated imaging of the block face often results in altered viscosity properties and electron-beam-induced damage to the plastic block, causing the block to become difficult to section. By reducing the primary beam-landing energy, we can reduce damage to sample. Moreover, the increase in signal to noise provided by the acceleration of the BSEs reduces the dose per unit area and allows for significantly faster scan rates at higher magnifications. The increase in signal to noise and the ability to operate at lower dose rates should not only benefit SBEM but also other techniques, such as FIB-SEM [8, 24, 25], which is limited by probe-penetration depth and detector sensitivity.

### Characterizing detector response as a function of backscatter electron energy

As detailed in the “Methods” section, above, the two backscatter detectors (Gatan BSD and FEI CBS) were configured differently. In the conventional backscatter detection mode without deceleration (Gatan BSD), the signal produced in the silicon photo-diode detector drops off significantly, as the accelerating voltage is lowered. To determine the rate at which the signal in each detector drops off as a function of electron energy, we measured the detector response when the electrons were backscattered from a gold substrate compared with a carbon background as the difference between gold and carbon signals.

The FEI Quanta FEG 200 microscope was aligned stepwise for accelerating voltages of 5–1-keV accelerating voltage, and a Faraday cup (Ted Pella, Inc.) measured the beam current at a fixed spot size for each voltage. By integrating the backscatter signal difference for the same area of the gold sample and carbon background at each accelerating voltage, we could normalize backscatter signal for beam-current differences between accelerating voltages. In addition, we normalized the signal according to the backscattering coefficient *η* for gold, where

2 $$\eta = \, N_{\text{backscattered}} / \, N_{\text{incident}}$$

is the ratio of the number of BSEs to the number of incident electrons. The backscattering coefficient is a function of the electron energy and is well established for gold [26]. Since we did not know the actual gains on the amplifiers or the relationship between detectors, we kept the amplifier gain and brightness settings fixed throughout the measurement process. The signal was determined as the difference between the gold sample and the carbon background.

**Fig. 6:**
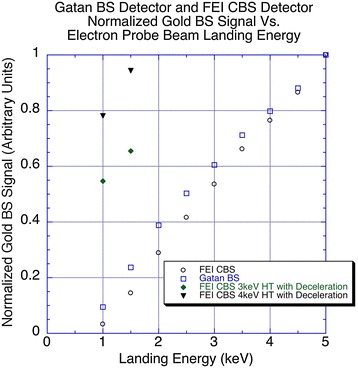
Normalized detector backscatter signal in arbitrary units for fixed detector gain and offset. The *open circles* show the normalized response of the FEI CBS detector as a function of electron energy without any sample biasing. The *open squares* show the normalized response of the Gatan backscatter detector as a function of electron energy without sample biasing. The *filled diamonds* show the normalized response of the CBS detector with 3-keV gun-acceleration voltage and deceleration voltages sufficient to achieve 1- and 1.5-keV landing energy. The *closed triangles* show the normalized response of the CBS detector for 4-keV acceleration voltage and deceleration voltages sufficient to achieve 1- and 1.5-keV landing energies. The results at 1-keV landing energy show an improvement of ×16.5 in signal for 3-keV column-electron energy and a ×23.6 increase in signal for 4-keV column-electron energy. The results at 1.5-keV landing energy show an improvement in backscattered signal of ×4.5 with 3-keV column-electron energy, and a ×6.5 increase in signal at 4-keV column-electron energy

Normalized results are given in Fig. 6 for both detectors on the same SEM, such that the 5-keV strong signal is set to unity. Because the two detectors have different configurations for detection, we set the values in Fig. 6 to unit-less values, so this graph should not be inferred to compare signal to noise between to the two detectors). Instead, it is intended to demonstrate a linear drop in signal to about 2.0 keV, at which point, the decreases become non-linear. This is presumably due to less penetration through the surface passivation layer on the silicon diodes of the Gatan BSD.

The results of both detector responses as a function of gun-accelerating voltage are also shown in Fig. 6. To compare the two detectors, we kept the amplifier gains and brightness settings constant for both detectors, and the central ring of the CBS was turned off.

The results show that depending on the gun-acceleration potential and the deceleration voltage, signal level increases of up to 20× and 6× or greater are possible at 1- and 1.5-keV landing energy, respectively, for decelerated probe-beam electrons versus the conventional SBEM. In addition, the signals in the CBS detector used with deceleration are actually higher than those where deceleration was not applied. We attribute this to the fact that the bias fields help collect and collimate the BSE electrons toward the BSD detector.

To demonstrate the increase in signal produced by the acceleration of BSEs when the reversal potential is used, we used the FEI CBS detector with the central ring turned off (the central ring becomes saturated with the SE signals and should be turned off when detecting BSEs with deceleration). The Gatan BSE detector was not used in this deceleration experiment, because the detector becomes saturated from accelerated SEs.

With the FEI CBS detector’s central ring turned off, we explored the BSE signals from the gold sample with 3- and 4-keV gun-accelerating energies and deceleration appropriate to achieve 1- and 1.5-keV landing energies. Although the electrons are decelerated when they travel down the column from the objective pole piece to the sample, electrons leaving the sample as BSEs are re-accelerated by the same amount. Therefore, the BSEs have nearly the same energy as the electron beam at the pole piece, theoretically with some energy loss due to the inelastic collisions with the atoms of the sample. For example, a 3-keV electron will be decelerated to a 1.5-keV landing energy in a −1.5-keV bias potential, and the BSEs will be re-accelerated to energy of almost 3 keV before striking the detector.

**Fig. 7:**
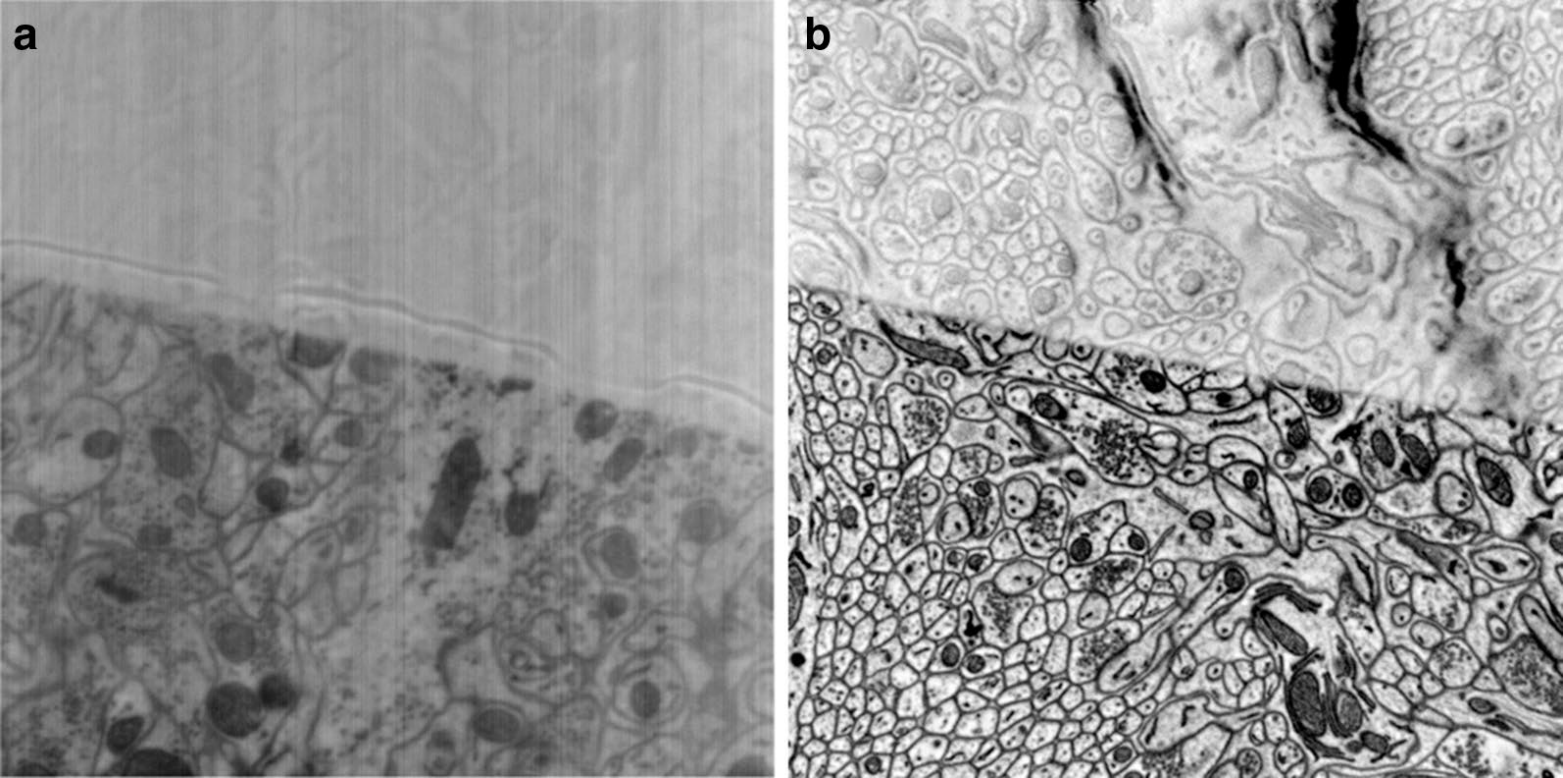
Comparison of two images of a cerebellum block with a 30-nm-thick blank plastic overlay acquired with 1.5-keV probe-beam-landing energy. **a** Image acquired with 1.5-keV high tension and no deceleration. **b** Image acquired with 3-keV high tension and −1.5-keV deceleration potential. Both images show a near equivalent penetration depth. However, **b** shows a significant improvement in signal as a result of BSE re-acceleration

Figure 7 compares the images acquired with and without beam deceleration. Both images were acquired with 1.5-keV landing energy. However, in the panel of Fig. 7b, a 3-keV column energy combined with a −1.5-keV deceleration bias results in a dramatic increase in signal to noise. In principle, as the column-electron energy is increased and the deceleration fields are increased, the backscatter signal should be significantly improved.

We explored a number of column-beam energies and deceleration potentials and found that the best results were obtained with deceleration between −1 and −2 keV and with a gun-accelerating energy of 2.5 keV and higher. For column-beam energies less than 2 keV, the beam current drops off quickly, and the image quality and stability are less than ideal. For deceleration voltages less than −1 keV, the electron re-acceleration was not as dramatic. For deceleration voltages higher than −2.5 keV, focus and astigmatism stability were decreased, and sample movements and distortions became problematic. For this geometry, we obtained our best results with moderate deceleration. However, we feel that, by careful design of the deceleration hardware and geometry, it should be possible to mitigate stability issues, enabling long runs at much higher bias voltages.

### Using the biasing field to spatially differentiate SE from BSE signals

The SBEM technique relies primarily on BSE signals for image generation. The image contrast arises from differences in electron scattering from the lighter Z-number atoms (hydrogen, carbon, nitrogen and oxygen) in the tissue and the plastic-embedding media, and the high-Z elements (osmium, uranium and lead) from the stains used in the specimen-preparation protocol. SEs are also created at the sample surface but usually have energies lower than 50 eV and, thus, are not detected in a photo-diode backscatter detector. Once deceleration is applied, these same low-energy secondary electrons are accelerated by the electric fields, striking the detector with sufficient energy to produce a measurable signal.

**Fig. 8:**
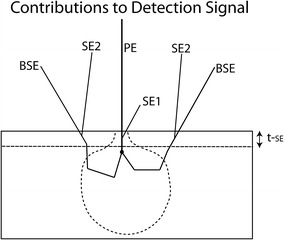
Relative contribution of backscattered and secondary electrons from the sample to the detection signal. Type SE1 electrons are created by interactions with the primary electron (PE) beam. Type SE2 electrons are created by interactions with the BSE. Low-energy SE electrons (<50 eV) typically have mean free paths between scattering events of 5–15 nm, depending on the sample composition, and do not escape from the sample unless they are produced extremely close to the specimen surface. BSEs can scatter out from deep inside the sample and be detected

In Fig. 8, we illustrate the various scattering mechanisms for detectable SEM signals. The primary electron-probe beam (PE) strikes the sample where it can produce secondary electrons near the surface with sufficient energy to escape the surface. The SEs produced by interaction with the primary electron are labeled SE1. A primary beam electron will typically scatter within the sample many times, producing additional SEs, which lack the energy to escape and be detected. If the PE is backscattered near the surface, it can create additional SEs that can escape the surface to be detected. These signals are labeled SE2. A third type of SE signal can be created through BSEs interacting with the chamber or pole-piece surface. In the conventional scanning electron microscopy, where the microscope is operated typically in the energy range of 5–30 keV, BSEs can scatter out of the sample from much deeper inside the substrate due to significantly longer mean free paths than the low-energy secondary electrons, and, as such, BSEs are considered to be lower resolution. In the figure, t-SE represents the depth from which 50 eV and lower-energy SEs can escape. For most materials, the depth from which SEs can escape is on the order of 5–15 nm. However, as the landing energies of the electrons are reduced to about 1 keV, the escape depth of the BSEs approaches a few tens of nanometer, similar to the SE signal [23], and, therefore, carries higher resolution information of the sample.

**Fig. 9:**
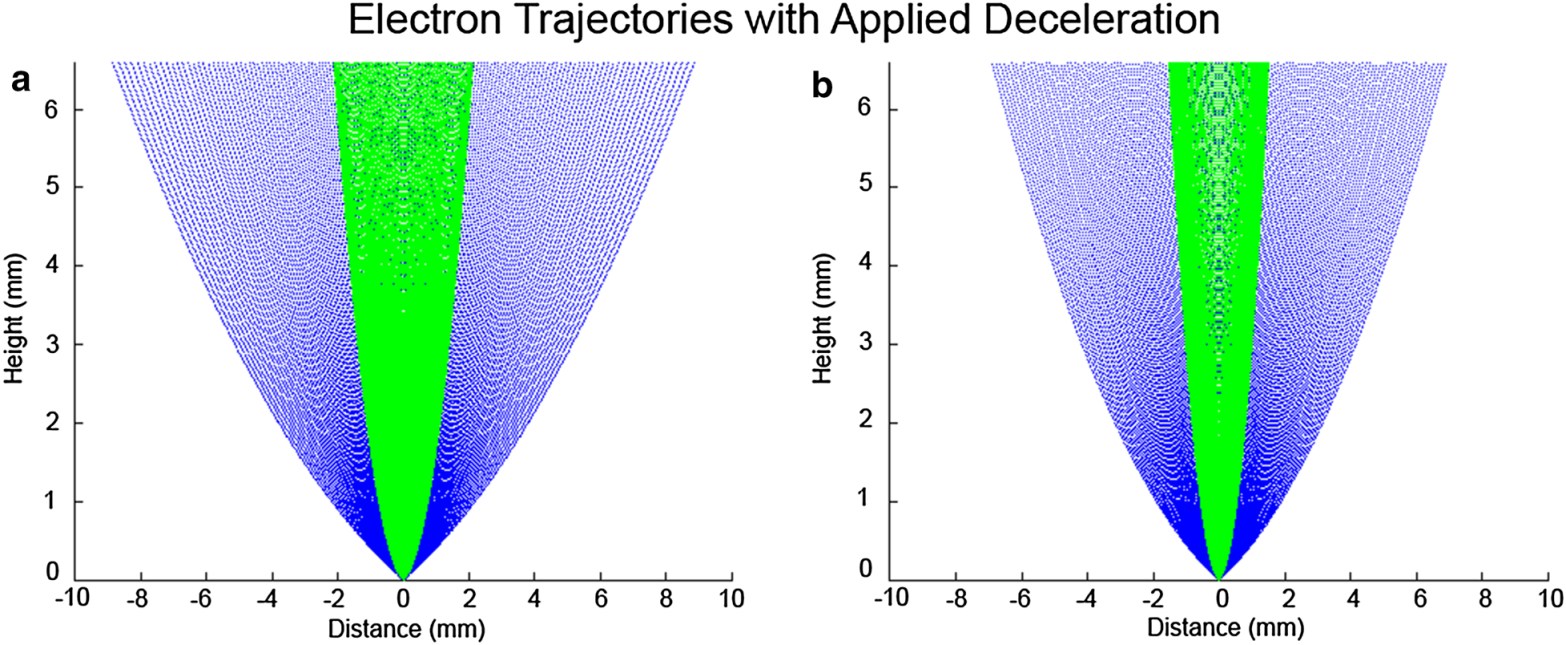
Calculation of the trajectories of the scattered electrons for BSEs (*blue*) and SEs (*green*) in a biasing field. Trajectories are plotted for electrons scattered from a point on the surface equally distributed through an angle of 0°–70° from the beam axis. **a** 3-keV gun-accelerating voltage with a −1.5-keV decelerating potential, resulting in a 1.5-keV beam-electron-landing energy. **b** 4.5-keV gun-accelerating voltage with a −3-keV decelerating potential, resulting in a 1.5-keV beam-electron-landing energy. The simulation matches well with the observed signal in the various rings of the CBS detector as a function of applied bias voltage

When imaging with the monolithic Gatan BSE detector and using deceleration voltages of −1 keV and greater, the SE signals dominated the images. Using the FEI CBS detector and looking at the signals in the individual concentric rings, we observed that these low-energy SEs, which are much more easily captured by the bias field, tend to be focused toward the center of the detector. The higher energy BSEs, by contrast, tend to be focused toward the outer regions of the backscatter detector. These observations are in agreement with the calculated trajectories of BSEs and SEs under the influence of a biasing field, as shown in Fig. 9, and are consistent with data presented elsewhere [27]. Using the configurable FEI CBS detector, we could separate the SE signal from the BSE signal by reading from the outer three rings for BSE signals, and the inner-most ring for SE signals. Figure 1c illustrates this setup as used in combination with deceleration via stage biasing.

**Fig. 10:**
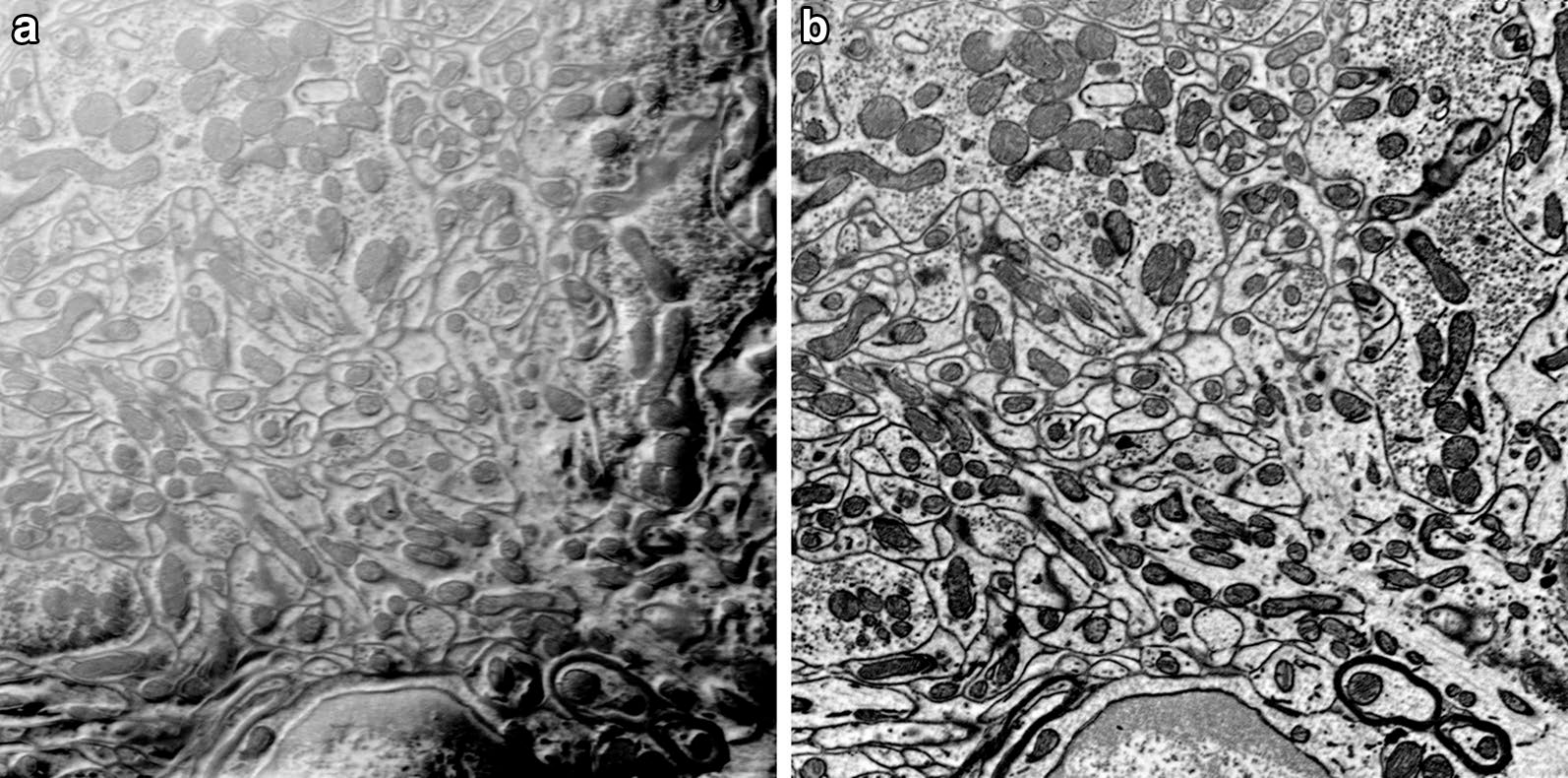
Typical images collected on the FEI CBS detector with 1.5-keV deceleration voltage. The separation of SE signals from BSE signals is possible by turning off the inner-most ring and using only the outer rings of the FEI CBS detector. Images were collected at 3-keV gun-accelerating electron energy and −1.5-keV deceleration voltage. **a** Signal from the central ring only. The signal is composed of a mix of BSEs and SEs, but it is dominated by SEs. **b** Signal from the outer three rings of the CBS detector. The signal contains almost no SE signal. With the monolithic Gatan BSE detector, separation of the BSE and SE signals with deceleration is not possible unless the SEs are blocked. The intensity has been inverted to produce TEM-like contrast

Figure 10 shows typical images collected with the CBS detector at 3-keV gun-accelerating voltage and −1.5-keV deceleration voltage. Figure 10a, using all rings of the detector, shows strong topographic contrast in addition to Z contrast, indicating that it is a mix of SE and BSE signals. In comparison, Fig. 10b, using only the outer three rings, shows only Z contrast with almost no topographic details, confirming that it is nearly a pure BSE signal image. The SE signal mixed with some BSE signal is clearly separated from the pure backscatter signal collected in the outer rings. Without this separation of BSE and SE signals, it would not be possible to use the deceleration techniques to lower electron-landing energy and simultaneously increase signal without obtaining charging type artifacts in the images, such as those shown in Fig. 10a. We attribute this charging artifact to the fact that low-energy SEs are easily affected by surface charging and the local electric fields created as a result of sample biasing. Enlarging the hole in the Gatan BSE detector or masking the center of the detector and using the deceleration fields to steer the SEs toward the center hole or mask on the detector will produce a similar separation of BSEs from SEs.

### Enhanced SBEM volume acquisition using deceleration

**Fig. 11:**
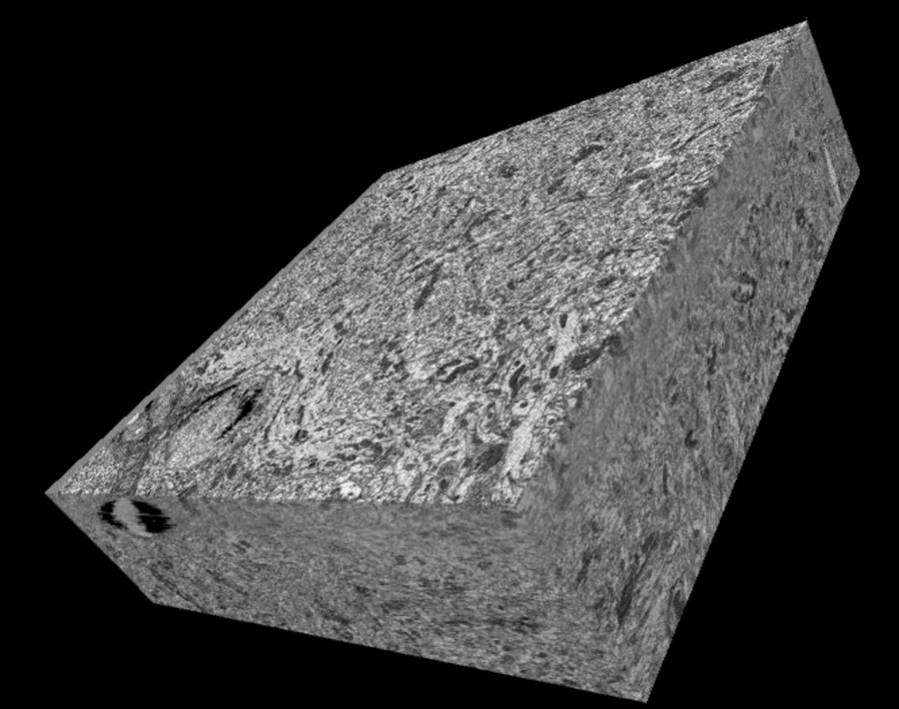
10 µm × 10 µm × 4 µm volume of rodent cerebellum collected by SBEM using sample biasing. Imaging was performed at 3.0-keV column-electron energy and a landing energy of 1.5 keV. The beam current was 129 pA at the sample with a magnification of ×3200 and 5 µs dwell time per pixel. The cutting depth on this sample was 50 nm

Using our improved deceleration protocol, we collected large-scale volume data sets using the 3View-equipped FEI Quanta FEG SEM. First, we wanted to ensure that the sample could be reliably cut with a large sample bias applied and the diamond knife sitting at ground. Figure 11 shows a reconstructed volume comprised of serial block-face images collected with 3-keV electron-probe energy and 1.5-keV landing energy. The ultramicrotome cuts were 50-nm thick. The images were collected at 3200× magnification and 5-µs dwell time.

### Adding an aperture to the monolithic BSD enabled the discrimination of BSEs and SEs similar to that of the CBS detector

With the ability to separate BSE and SE signals by turning off the inner ring and using only the outer rings of FEI’s concentric-ring BSE detector in combination with moderate deceleration, we then took the next step to determine if a simplified setup could be used, employing an monolithic Gatan BSE detector with a blocking aperture to remove the large SE signal from our images.

Careful design of the correct blocking aperture diameter for the geometry and the deceleration potential is critical for BSE/SE separation. We found that the most important parameters were the gun-accelerating voltage, decelerating voltage, and landing energy of the probe beam; the distance from the sample to the detector; and the diameter of the SE-blocking aperture.

Weighing these considerations, we built a simple prototype blocking aperture by modifying a standard TEM aperture, which had a 5.0-mm outer diameter ring with a central hole of 0.8-mm diameter. This custom SE-blocking aperture was centered and attached above the diode to the grounded shielding of the Gatan BSE detector. Using this setup, any SEs striking the aperture should be conducted away quickly, leaving only BSEs to be detected on the BSE device.

**Fig. 12:**
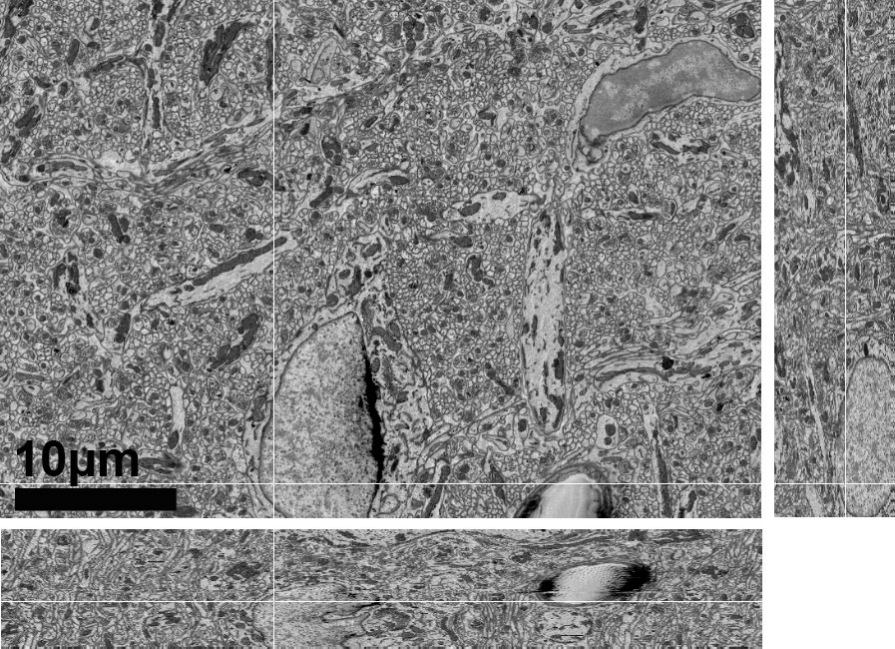
3D cross-section view of a 40 µm × 40 µm × 8 µm volume of rodent cerebellum collected by SBEM using 3-keV primary beam energy and a −1-keV sample bias to achieve a 2-keV landing energy. The volume was collected on a Zeiss Sigma using the standard monolithic BSE detector from Gatan and a 5-mm outer diameter and 0.8-mm inner diameter SE-blocking aperture appropriate to blocking SEs from a 3-keV primary beam, a −1-keV bias potential, and an 8.9-mm working distance. The volume was collected at 1.5K× magnification, 1.5-µs dwell time, and a 4K × 4K× raster scan at a beam current of 55 pA

As proof of principle, we tested this setup with a 3-keV column energy and a −1.0-keV decelerating potential between the pole piece and the sample (8.0-mm working distance). Figure 12 shows a 2D slice through a volume collected on the 3View-equipped Zeiss Sigma using this setup. The images remained quite stable during the cutting and imaging process. 150 sections were cut at 60-nm thickness at 1.5-µs dwell times, 55-pA beam current, 1.5K× magnification, and a 4K × 4K raster.

The addition to Gatan’s microscopy suite of a new autofocus and autostigmation routines should enable stable imaging for weeks at a time using this technique. Future development of deceleration-based detection would also benefit from fabrication of a variety of blocking apertures to better optimize aperture geometry to the deceleration potential employed.

## Conclusion

This study demonstrates that using beam deceleration in the SBEM results in higher quality images. Thinner axial-penetration depth achieved with probe-beam deceleration improved axial resolution. A remarkable increase in total signal collection of 20-fold or higher was produced with the use of relatively low-deceleration potentials as compared with the conventional SEM imaging using the same landing energies. This increase in signal allowed for much lower interrogation currents on the sample, resulting in improved sample-sectioning properties.

Here, we demonstrated a procedure that mitigates the degradation of the image quality resulting from low-energy SEs when using deceleration. By removing SEs from the images, using the FEI concentric backscatter detector or a simple SE-blocking aperture in a monolithic detector, beam deceleration becomes a feasible approach to achieve limited penetration depths with sufficient sample and imaging stability to produce large volumes of data. Additional improvements in sample preparation, particularly the use of conductive epoxy resins formulated for SBEM, should improve the technique of beam deceleration by producing uniform electric fields at the sample surface and reducing surface-charging effects.

We believe that this study demonstrates the first of many improvements that can be made to SBEM imaging. We observed drift in image position, astigmatism, and focus changes, so these artifacts still represent challenges that need to be addressed to optimize volume quality. We found, however, that these were not significant obstacles at lower deceleration voltages, and we now believe that, with improved system geometry and the addition of autofocus software now available from Gatan, these instabilities can be addressed easily, enabling significant improvements in volumetric reconstructions.

## Abbreviations

SBEM: serial block-face scanning electron microscopy
BSE: backscattered electron
BSD: backscattered electron detector
SE: secondary electrons

## References

[1] Shu X, Lev-Ram V, Deerinck TJ, Qi Y, Ramko EB, Davidson MW, Jin Y, Ellisman MH, Tsien RY (2011). A genetically encoded tag for correlated light and electron microscopy of intact cells, tissues, and organisms. PLoS Biol.

[2] West JB, Fu Z, Deerinck TJ, Mackey MR, Obayashi JT, Ellisman MH (2010). Structure-function studies of blood and air capillaries in chicken lung using 3D electron microscopy. Respir Physiol Neurobiol.

[3] Williams ME, Wilke SA, Daggett A, Davis E, Otto S, Ravi D, Ripley B, Bushong EA, Ellisman MH, Klein G, Ghosh A (2011). Cadherin-9 regulates synapse-specific differentiation in the developing hippocampus. Neuron.

[4] Jurrus E, Hardy M, Tasdizen T, Fletcher PT, Koshevoy P, Chien CB, Denk W, Whitaker R (2009). Axon tracking in serial block-face scanning electron microscopy. Med Image Anal.

[5] Nguyen JV, Soto I, Kim KY, Bushong EA, Oglesby E, Valiente-Soriano FJ, Yang Z, Davis CH, Bedont JL, Son JL, Wei JO, Buchman VL, Zack DJ, Vidal-Sanz M, Ellisman MH, Marsh-Armstrong N (2011). Myelination transition zone astrocytes are constitutively phagocytic and have synuclein dependent reactivity in glaucoma. Proc Natl Acad Sci USA.

[6] Leighton SB (1981). SEM images of block faces, cut by a miniature microtome within the SEM—A technical note. Scan. Electron Microsc..

[7] Denk W, Horstmann H (2004). Serial block-face scanning electron microscopy to reconstruct three-dimensional tissue nanostructure. PLoS Biol.

[8] Knott G, Marchman H, Wall D, Lich B (2008). Serial section scanning electron microscopy of adult brain tissue using focused ion beam milling. J. Neurosci.

[9] Ohta K, Sadayama S, Togo A, Higashi R, Tanoue R, Nakamura K (2012). Beam deceleration for block-face scanning electron microscopy of embedded biological tissue. Micron.

[10] Bauer E (1985). The resolution of the low-energy electron reflection microscope. Ultramicroscopy.

[11] Telieps W, Bauer E (1985). An analytical reflection and emission Uhv surface electron-microscope. Ultramicroscopy.

[12] Frank L, Mullerova I (1999). Strategies for low- and very-low-energy SEM. J Electron Microsc.

[13] Matsuda K, Ikeno S, Mullerova I, Frank L (2005). The potential of the scanning low energy electron microscopy for the examination of aluminum based alloys and composites. J Electron Microsc.

[14] Mullerova I (2001). Imaging of specimens at optimized low and very low energies in scanning electron microscopes. Scanning.

[15] Khursheed A, Osterberg M (2004). A spectroscopic scanning electron microscope design. Scanning.

[16] Pluk H, Stokes D, Lich B, Wieringa B, Fransen J (2009). Advantages of indium-tin oxide-coated glass slides in correlative scanning electron microscopy applications of uncoated cultured cells. J Microsc.

[17] Titze B, Denk W (2013). Automated in-chamber specimen coating for serial block-face electron microscopy. J Microsc.

[18] Kanaya K, Okayama S (1972). Penetration and energy-loss theory of electrons in solid targets. J Phys D Appl Phys.

[19] Joy DC (1995). Monte carlo modeling for electron microscopy and microanalysis.

[20] Goldstein JI, Newbury DE, Echlin P, Joy DC, Lyman CE, Lifshin E, Sawyer L, Michael JR (2003). Scanning electron microscopy and microanalysis.

[21] Reimer L, Hawkes PW (1998). Scanning electron microscopy—physics of image formation and microanalysis. Springer series in optical sciences.

[22] Agar, A.W., Alderson, R.H., Chescoe, D.: Principles and practice of electron microscope operation, In: Glauert, A.M. (ed.) American Elsevier Publishing Co, New York (1974)

[23] Joy DC, Joy CS (1996). Low voltage scanning electron microscopy. Micron.

[24] Helmstaedter M, Briggman KL, Denk W (2008). 3D structural imaging of the brain with photons and electrons. Curr Opin Neurobiol.

[25] Schroeder-Reiter E, Perez-Willard F, Zeile U, Wanner G (2009). Focused ion beam (FIB) combined with high resolution scanning electron microscopy: a promising tool for 3D analysis of chromosome architecture. J Struct Biol.

[26] Assa’d AMD, El Gomati MM (1998). Backscattering coefficients for low energy electrons. Scanning Microsc.

[27] Phifer D, Tuma L, Vystavel T, Wandrol P, Young RJ (2009). Improving SEM imaging performance using beam deceleration. Microsc Today.

